# Developing a Robust Fuzzy Inference Algorithm in a Dog Disease Pre-Diagnosis System for Casual Owners

**DOI:** 10.3390/ani14243561

**Published:** 2024-12-10

**Authors:** Kwang Baek Kim, Doo Heon Song, Hyun Jun Park

**Affiliations:** 1 Department of Artificial Intelligence, Silla University, Busan 46958, Republic of Korea; gbkim@silla.ac.kr; 2 Department of Computer Games, Yong-In Art & Science University, Yongin 17145, Republic of Korea; mypham@hanmail.net; 3 Division of Artificial Intelligence Engineering, National Korea Maritime & Ocean University, Busan 49112, Republic of Korea

**Keywords:** pet dog disease, pre-diagnosis, multi-layered fuzzy inference, PFCM, robustness

## Abstract

While the pet market is continuously rapidly increasing in Korea, pet dog owners feel uncomfortable in coping with pet dog’s health problems in time. In this paper, we propose a pre-diagnosis system based on neuro-fuzzy learning, enabling non-expert users to monitor their pets’ health by inputting observed symptoms. To develop such a system, we form a disease–symptom database based on several textbooks with veterinarians’ guidance and filtering. The system offers likely disease predictions and recommended coping strategies based on fuzzy inference. We evaluated three fuzzy inference algorithms—PFCM-R, FHAL, and MNFL. While PFCM-R achieved high accuracy with clean data, it struggled with noisy inputs. FHAL showed better noise tolerance but lower precision. PFCM-R is a variant of well-known fuzzy unsupervised learner FCM, and FHAL is the hybrid fuzzy inference engine based on Fuzzy Association Memory and a double-layered FCM we developed. To make the system more robust, we propose the multi-layered neuro-fuzzy learner (MNFL) in this paper, which effectively weakens the association strength between the disease and the observed symptoms, less related to the body part on which the abnormal symptoms are observed. In experiments that are designed to examine how the inference system reacts under increasing noisy input from the user, MNFL achieved 98% accuracy even with non-erroneous inputs, demonstrating superior robustness to other inference engines. This system empowers pet owners to detect health issues early, improving the quality of care and fostering more informed interactions with veterinarians, ultimately enhancing the well-being of companion animals.

## Simple Summary

As global pet ownership rises, managing the health of aging dogs has become a growing concern, particularly due to the high costs and challenges in recognizing early symptoms. In Korea, many casual pet owners struggle to identify abnormal behaviors and address them effectively without expert knowledge. Thus, we propose a pre-diagnosis system for casual dog owners that infers possible diseases from queries based on observed abnormal symptoms. This proposed system, based on a fuzzy inference algorithm, is not to aiming to compete with veterinarians but to alert the casual pet caregiver to monitor abnormal behavior indicative of disease, to be addressed as early as possible. The experimental results verify that the proposed system is sufficiently robust under noisy input, which is very common when non-expert casual owners use this system.

## 1. Introduction

The global pet market is continuously growing worldwide, valued at $235 billion in 2022, and this trend will be maintained, since its compound annual growth rate is estimated at 7.5% for the next ten years [1]. In the US, over 70% of households are pet owners and over 46% of households (65.1 million households) own at least one dog [2]. The US report also pointed out that, since 32% of pet owners are millennials, such a trend will be maintained for a period of time, and dog owners spend $730 annually per dog. Based on the information provided by petinsurer.com, American dog owners spend on average $56 per month per dog for unlimited coverage of pet health insurance, since the cost of treating common diseases for older dogs, such as diabetes, glaucoma, and hip dysplasia, costs $5000–$10,000 without insurance coverage [3].

In Korea, a recent nationwide pet survey reported that over 30% of households own a pet and 25% of total households own at least one dog, and this trend is growing fast [4]. According to that report, Korean dog owners spent over $1000 per dog in 2019, which continues to increase. The high cost of caring for the pet dog is partly due to the high costs of high-quality care products that include beauty care, special snacks, and premium toys [5], but the healthcare cost for an old dog (over 10 years old) is also a major concern [4].

According to the survey of 1000 Korean dog owners reported in [4], the top three difficulties in raising a pet dog are ‘coping with deteriorating health problems’, ’caring details’, and ‘high caring cost’. Owners who have dogs older than 10 years (constituting about 20% of dog owners) feel especially frustrated, because they do not know what the cause of the pet’s discomfort is, or how to cope with it. The three most common types of disease that occur in old dogs are eye-related, skin-related, and oral health-related diseases [4].

With limited knowledge of dog diseases, casual owners tend to fail to detect diseases or rely on unscientific methods to manage their pet dog’s abnormal behavior [6]. What they need the most is not a professional expert system designed for veterinarians [7,8,9,10], but a first-hand abnormality monitoring tool that can cover a wide range of diseases based on the symptoms of the pet that the owner can detect, and the system should provide helpful coping strategies without requiring deep knowledge of the diseases [11]. The role of such a pre-diagnosis information system is not to compete with veterinarian nor expert systems, which should maintain high accuracy with medical test data for correct diagnosis. Rather, the system is designed to alert the casual pet caregiver to monitor abnormal behavior indicative of disease, to be addressed as early as possible.

Such self-controllable pre-diagnosis software can enhance the trust between pet owners and veterinarians, since pet owners can understand what such abnormal behavior means before consulting with veterinarians, and it is confirmed that Korean pet owners usually go through several verification steps to verify the reliability of medical information obtained from the internet/YouTube and through consultation with veterinarians [12].

To design such pre-diagnosis system, we need a standardized symptom–disease database [10] and robust fuzzy inference system [13,14,15,16].

Unfortunately, there is no internationally recognizable dog disease database, nor has the Korean government approved a database for that purpose. Thus, we should construct such a database based on textbooks with veterinarians’ guidance, which will be explained in Section 2.1.

For the inference system based on the observed symptoms by casual owners, we adopt a fuzzy logic-based system, since the observed symptoms are not obtained from any medical test result nor any medical equipment. This is a clear distinctive characteristic of the pre-diagnosis system we aim to develop from the expert system, with rules from veterinarians that can utilize medical test results and expert knowledge.

Under such an environment, the inference system we develop can only use the association between input symptoms from the novice owner and the set of diseases defined in the database we construct. Thus, the inference system should use unsupervised learning since there are no available clinical test results from such inputs. For this reason, we decided to explore fuzzy logic-based inference algorithms.

Many different types of fuzzy logic-based inference are technically feasible for user input data through observation, but the target pre-diagnosis system controlled by casual pet owners must have robustness for real-world application. Since casual owners are not trained like medical staff, and casual pet owners’ observations are not based on any clinical data, part of the symptoms input by such users may result from incorrect observation. A survey conducted in India revealed that no more than 30% of respondents were aware of nine common dog diseases when asked, other than stating that rabies can be indicated by apparent aggressive behavior [7].

Also, when the pet dog has a compound disease, the untrained owner may believe that all observed symptoms are related to a single disease, thus providing unrelated symptoms for one target [16]. Positing a query to the symptom–disease association database with such irrelevant/incorrect symptoms will negatively influence the strength of inference. Thus, the robustness of the inference system is important in real-world application. Thus, the inference system we develop should be robust, in that the inference system should be sufficiently immune to erroneous input from casual owners.

In this paper, we evaluated three fuzzy inference algorithms—PFCM-R, FHAL, and MNFL PFCM-R (Possibilistic Fuzzy C-Means with Regularization) [17] is a variant of the well-known fuzzy unsupervised learner FCM, considered noise-insensitive among the FCM family. FHAL (Hybrid Fuzzy Association Learning) is the hybrid fuzzy inference engine we developed, based on Fuzzy Association Memory [18] and double-layered FCM [16]. FHAL uses both direct association and indirect association to mitigate the negative influence of erroneous input. To make the system more robust, we propose the multi-layered neuro-fuzzy learner (MNFL) in this paper, which effectively weakens the association strength between the disease and the observed symptoms, less related to the body part on which the target disease can appear. In the experiment, we created a noisy environment to test each algorithm’s robustness against inputs containing erroneous input symptoms.

The structure of this paper is as follows: In Section 2.1, we will explain the symptom–disease database, constructed based on several textbooks with filtering from veterinarians. Three different fuzzy inference algorithms—Possibilistic Fuzzy C-Means with Regularization (PFCM-R) [17], Fuzzy Hybrid Association (FHAL) [16], and the newly designed multi-layered neuro-fuzzy learning method (MNFL)—will be explained in Section 2.2, followed by the performance results among three fuzzy inference algorithms for robustness against noisy input in Section 3.

## 2. Materials and Methods

### 2.1. Symptom–Disease Database Construction

Our symptom–disease association database is formed with three main tables, such as SymptomT (SymptomID, Description, ObservedPart), DiseaseT (DiseaseID, Description, AppearedPart), and Association (ID, DiseaseID, SymptomID), as well as two auxiliary tables that define the time at which the user observes an abnormality (ObservedPart Table) and the time at which the disease actually appears with respect to the textbook’s classification (AppearedPart Table).

From the referenced textbooks [19,20,21,22], we derived 241 symptoms and 249 diseases that occur relatively frequently, as well as symptoms that can be observed by casual users under multiple veterinarians’ guidance and filtering. However, we used only 50 diseases that have more than five symptoms in the database for the experiment in this paper, detailed in Section 4, to test the robustness under a noisy input environment.

The field ObservedPart in Table SymptomT represents where the user observed such a symptom from the ObservedPart Table. It consists of 16 parts such as eye, nose, ear, and feces. This field is for the user interface (UI) of the software, in that the user selects the observed part first and then the system shows observable symptoms to the user to select the most similar symptom that they observed from the pet dog.

The field AppearedPart consists of 12 body parts on which the disease could appear, such as the skin, eyes, ears, and circulatory system with respect to the referenced textbooks’ classification from the AppearedPart Table.

Thus, we may state that the ObservedPart attribute is for casual user’s convenience and the AppearedPart attribute is for correct inference, used among veterinarians.

The structure of each database table is configured as shown in Table 1.

Then, the proposed system works as follows.

**Figure 1:**
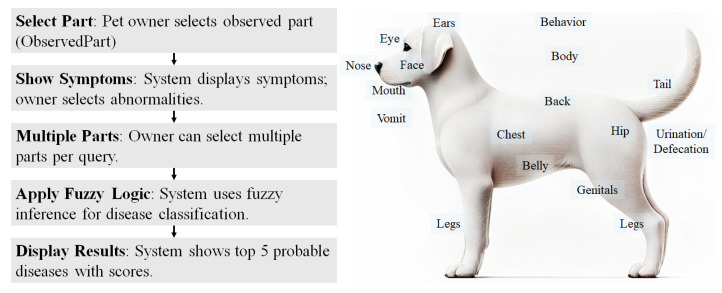
The workflow of the inference system and ObservedPart selection interface.

(a) The pet owner is asked to select the specific part on which the observation is made first (ObservedPart), shown as in Figure 1.
(b) Then, the system suggests possible symptoms and the owner selects several observations, indicating that they saw “abnormality” regarding the dog.
(c) The owner can select more than one part per query.
(d) The system applies fuzzy inference algorithms with respect to the AppearedPart information, which is used for disease classification carried out by veterinarians from the textbooks we used.
(e) For each query, the system outputs the five most probable (related) diseases with fuzzy inference scores.

### 2.2. Fuzzy Inference Algorithms

#### 2.2.1. Possibilistic Fuzzy C-Means with Regularization (PFCM-R)

Fuzzy C-Means (FCM) is the most popular fuzzy unsupervised learner, but its noise sensitivity is a well-known weak point. Possibilistic C-Means (PCM) relaxes the probabilistic constraint of FCM to overcome that disadvantage and interprets the membership value as how typical a data object is for a given cluster [23]. However, the PCM algorithm often generates coincident clusters due to the initialization sensitivity. PFCM [24] combines the objective functions of these two algorithms to mitigate such known drawbacks.

While often generating better results than FCM, PFCM requires more data than FCM to obtain a stable solution, due to the complex objective function. The regularization of PFCM (PFCM-R) [17] flattens the solution space to obtain a similar solution that is relatively noise-insensitive and applicable with a small data size. Thus, we decided to implement PFCM-R in this application, and the details of objective function and update rules can be found in [17].

#### 2.2.2. Hybrid Fuzzy Association Learning (FHAL)

FHAL is motivated by Fuzzy Association Memory [18] and double-layered FCM [25], which have recently achieved decent results in the medical domain.

In real-world situations, a casual owner’s wrong observation may include unrelated symptoms when forming a single query to the system. A similar situation may occur when the dog has a compound disease; the casual owner may generate single query based on the misbelief that all those observed symptoms are related to single disease. In such cases, the strength of inference is negatively influenced by such outlier symptoms in the input.

**Figure 2:**
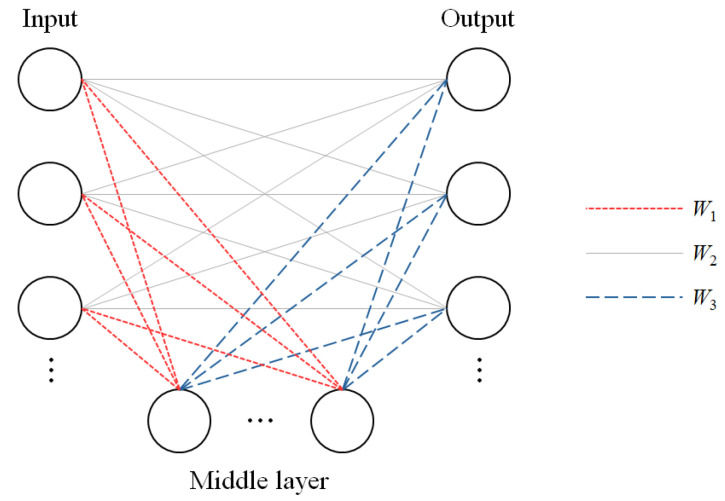
The fuzzy hybrid association learner structure.

Thus, it is important to exclude or at least mitigate the effect of such outlier input symptoms from the inference process. To accomplish this goal, we provide a double-layered structure, as shown in Figure 2.

As shown in Figure 2, there are three layers. The input layer consists of user input symptoms, and the middle layer represents the pet’s body parts to which input symptom is related, and the output layer consists of all potential diseases.

Throughout this paper, *w*_1_, *w*_2_, *w*_3_ refers to the weight matrices of relationship between input and middle layer, middle layer and output layer, and the direct association of input and output layer. The details of computing *w*_1_, *w*_2_, and *w*_3_ are fully explained in [16].

The role of the first association learning between the input layer and the middle layer is to mitigate the negative effect of outliers/unrelated input in the inference. Only patterns that have a relatively high association in this first learning cycle act as the input of the second FCM structure between the middle layer and the output layer, which are formed with the suggested diseases.

In the FHAL structure, the middle layer consists of 12 parts/behaviors, as shown in the database table AppearedPart in Figure 2.

#### 2.2.3. Multi-Layered Neuro-Fuzzy Learning (MNFL)

**Figure 3:**
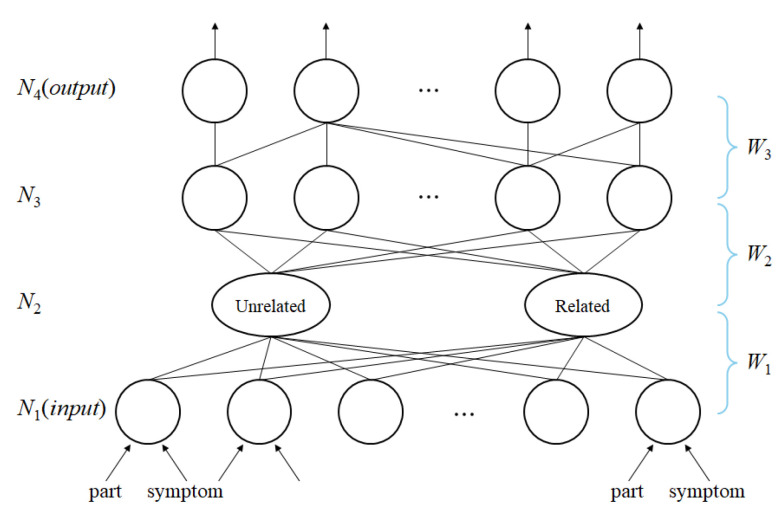
Multi-layered neuro-fuzzy learning.

In this paper, we propose a new fuzzy inference engine for robust reasoning. The motivation of using the multi-layered structure shown in Figure 3 is to filter the irrelevant observed symptoms (input pattern) with respect to the AppearedPart attribute where the disease can appear. Layer *N*_1_ in Figure 3 explicitly penalizes such erroneous input. The overall structure of the proposed multi-layered neuro-fuzzy learning (MNFL) structure is as shown in Figure 3.

**Figure 4:**
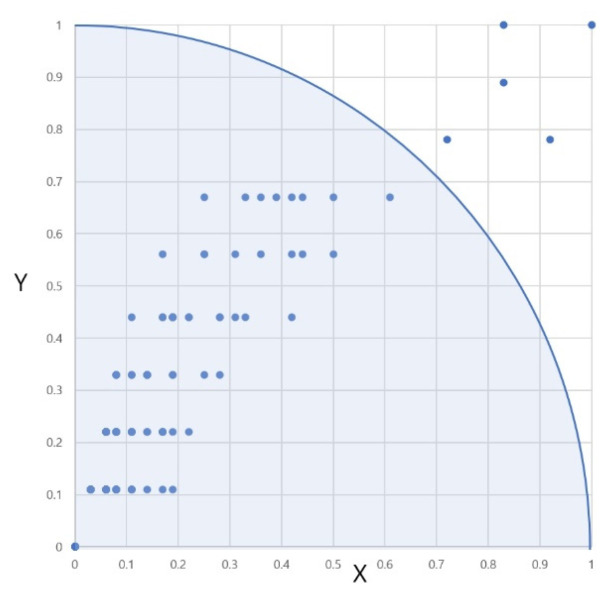
Connection strength *W*_1_.

In layer *N*_1_, which consists of all symptom nodes, the user provides the (observed part, observed symptom) pair as the input and only those nodes are activated and associated with two clusters of the *N*_2_ layer. Those two clusters (related/unrelated) can be understood as the coordinates (0, 0) and (1, 1) of Figure 4. *W*_1_ is the association strength between layer *N*_1_ and *N*_2_.

There are three weight parameters acquired from this structure.

In Figure 4, the *X* axis represents the normalized number of diseases that have the given symptom, and the *Y* axis represents the normalized number of AppearedParts of the disease that contains the given symptoms.

The centroid of the ‘Related’ cluster is (0, 0), meaning that the lower the number of diseases with the given symptom, the lower the number of AppearedParts of the disease, the input given by the user is closely related to the associated disease in fuzzy relationship. Equation (1) computes *W*_1_ membership strengths as below.

(1) $$u_{k}=\frac{1}{\sum_{j=1}^{c}{\left(\frac{d_{1k}}{d_{jk}}\right)}^{\frac{2}{m-1}}}\;w_{k}=u_{k}\;y_{k}=w_{k}\times x_{k}$$

where *u_k_* is the membership degree to the two clusters (*c* = 2), *m* is the fuzzy constant for *k* number of symptoms, *d* is the Euclidean distance between two centroids and the data, *y* is the output, and *x* is the input pattern.

**Figure 5:**
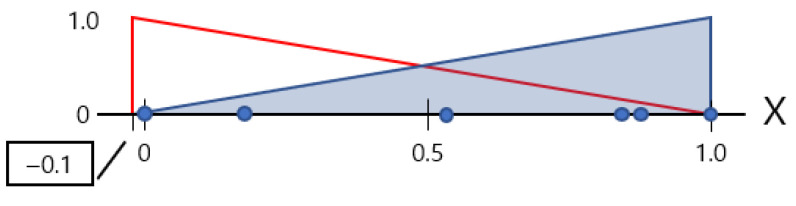
Connection strength W_2_.

The hidden layer *N*_3_ consists of all diseases in the database and *W*_2_ is the connection strength between *N*_2_ and *N*_3_ as shown in Figure 5.

The *X* axis of Figure 5 represents the normalized number of diseases that contain the input symptom of *N*_2_ with the same AppearedPart of the disease. *W*_2_, the membership strength, is computed by Equation (2) as follows.

(2) $$u_{k}=\frac{1}{\sum_{j=1}^{c}{\left(\frac{d_{1k}}{d_{jk}}\right)}^{\frac{2}{m-1}}}\;w_{k}=\left\{\begin{array}{c} u_{k}\;:\;u_{k}>0\\ -0.5\;:u_{k}=0 \end{array}\right.$$

where there are *c* disease clusters and *k* symptoms; *d* is the Euclidean distance between *c* cluster centroids and the *k*-th symptom with fuzzy constant *m*. We set *w_k_* = −0.5 when the membership degree is zero to penalize such a case in which that the body part that the user provided is not the same as the part on which the disease appears.

Then, the output of the *N*_3_ is computed as follows in Equation (3).

(3) $$y_{z}=\frac{1}{n}\sum_{i=0}^{k}w_{i}\times x_{i}$$

The hidden layer *N*_4_ consists of all target diseases in the database, and *W*_3_ is the connection strength between *N*_3_ and *N*_4_, as shown in Figure 3.

**Figure 6:**
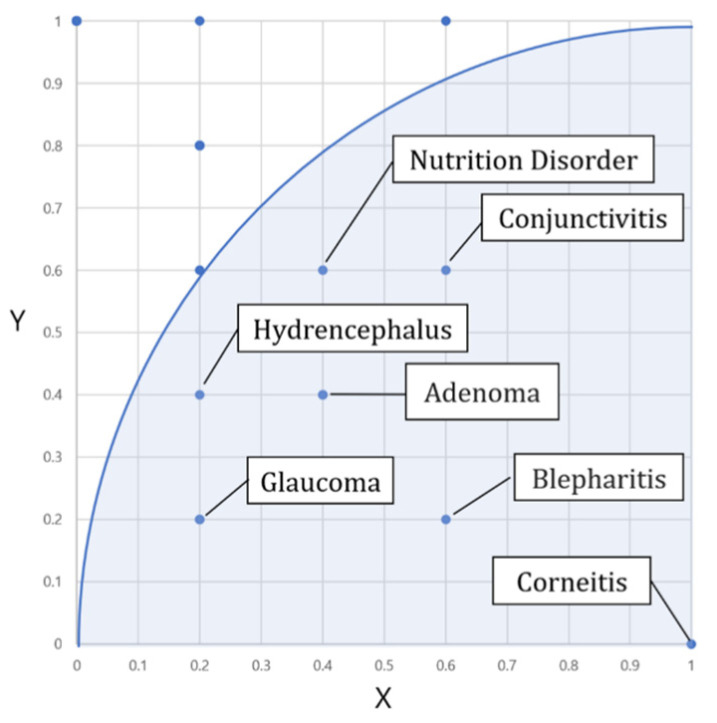
Connection strength W_3_ and relative distance of diseases.

Figure 6 is an example where the target disease is corneitis, displaying how other related diseases are positioned in this coordinate system. *W*_3_ denotes the relationship between the learned disease and the target disease.

In the coordinate system of Figure 6, the target disease is located at (0, 1) and the *X* axis represents the normalized number of common symptoms between the disease and the target disease.

The *Y* axis of Figure 6 represents the normalized difference in the number of symptoms between the target diseases and investigating disease. Then, the membership strength *W*_3_ and the final output will be computed as follows in Equation (4).

(4) $$u_{z}=\frac{1}{\sum_{j=1}^{c}{\left(\frac{d_{1z}}{d_{jz}}\right)}^{\frac{2}{m-1}}}\;w_{z}=u_{z}\;y_{z}=\frac{1}{n}\sum_{i=0}^{z}w_{i}\times x_{i}$$

where *n* is the number of activated disease nodes.

## 3. Results

All algorithms explained in this paper are implemented in Visual Studio 2022 with Intel(R) Core (TM) i7-8700 6-Core CPU, 32.0GB RAM PC. The symptom–disease database contained fifty different diseases that have at least 5 registered symptoms from 241 observable symptoms.

We compare the MNFL proposed in this paper with the hybrid association-based FHAL and the original PFCM-R-based implementation [17].

To compare the robustness of fuzzy inference, we provide 5 relevant symptoms for each target disease first, which defines the Noise0 set. Then, we change one input symptom randomly that is not related to the target disease (Noise1). Then, we add another noise by replacing the relevant symptom with noise to make the Noise2 and Noise3 sets. Thus, in the Noise3 set, there are two relevant symptoms and three irrelevant symptoms for each algorithm.

(1) Target diseases: 50 diseases that have at least five meaningful symptoms.
(2) Test Set: Noise0 contains five meaningful symptoms.

Noise1 has 1 noise symptom and 4 meaningful symptoms.

Noise2 has 2 noise symptoms and 3 meaningful symptoms

Noise3 has 3 noise symptoms and 2 meaningful symptoms. 

The accuracy in this experiment is defined as “correct” if the tested algorithm finds the target disease as the most probable disease under the given input; otherwise, it is “incorrect”.

The first performance measure is the accuracy over four data sets (Noise0, Noise1, Noise2, Noise3) in that the inference succeeds only when the target disease is the first output of the tested algorithm.

**Figure 7:**
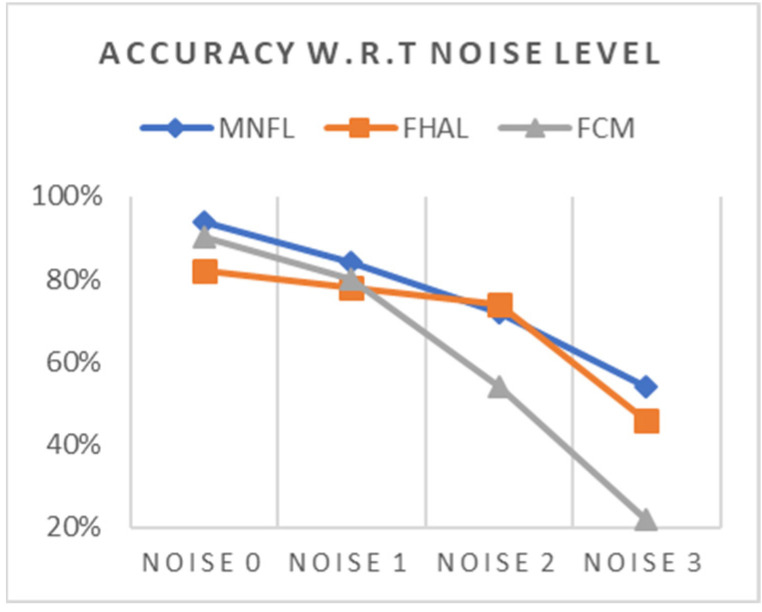
Accuracy trend over noise level.

Table 2 and Figure 7 summarize the accuracy test results.

As expected, PFCM-R (represented as FCM in the graph) is noise-sensitive, and the trend becomes unacceptable in the Noise2 and Noise3 sets. FHAL is relatively noise-tolerant, but its performance is worse than FCM in the Noise0 set. Thus, MNFL is the best of three in all test data sets, meaning that it is more accurate and robust at the same time.

**Figure 8:**
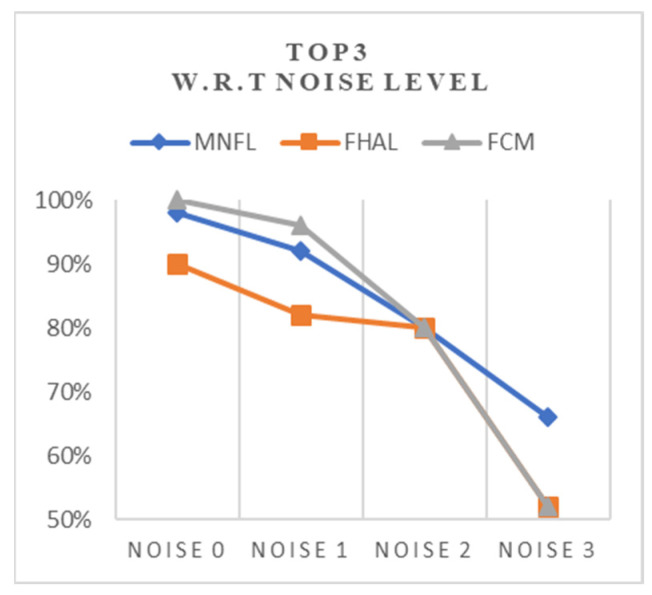
Top-three inference trend over noise level.

However, this self-controlled pre-diagnosis system does not use any medical data but relies on the casual owner’s observation only. Thus, when the owner feels the pet needs thorough examination, it may be acceptable if the inference system has a near-miss; that is, the real target disease is one of the top three possible diseases. Let us say that this is a ‘similar’ diagnosis. Thus, if we relax the accuracy metric to the rate the tested algorithm finds the target disease as a top three probable disease under a given input, the result with this ‘top 3 probable’ accuracy is summarized in Table 3 and Figure 8 as follows.

Interestingly, PFCM-R scores 100% when all relevant symptoms are given. However, PFCM-R fails when the number of noises is two or more. Our proposed MNFL algorithm shows a 4–12% more acceptable result than that of Table 2.

## 4. Discussion

Since our goal is to develop a pre-diagnosis system that relies on observed symptoms indicating that casual pet owners sense abnormality in their dog’s behavior, the inference algorithm must be sufficiently robust or immune to erroneous input. The experiment in this paper is designed to test that robustness under noisy input. In our pilot study period, casual dog owners tend to indicate an erroneous input rate of 1.7 on average when they are asked to select 5 symptoms for the target disease they had experienced.

Among the three fuzzy inference algorithms in the experiment, PFCM-R is a baseline competitor that is one of the most robust variants of the FCM family, in that its combined objective function enables to mitigate the well-known pitfalls of FCM and require not too much data with regularization effect. As expected, PFCM-R is strong when all relevant information is given (Noise0 Set). However, the inference accuracy quickly falls as the noise information is increased, especially in the Noise2 and Noise3 sets.

The second fuzzy algorithm, FHAL, is a hybrid model of Fuzzy Association Memory (FAM) and double-layered FCM that penalizes irrelevant input patterns that wrongly designated the dog’s body part on which the abnormality appears. However, the weakness of the FAM model is emphasized when almost all relevant information is given (Noise0, Noise1); that is, its inference power is not that strong, even though it has some level of robustness.

The proposed inference engine MNFL has a layer that is concerned with the relevance of the part information in the input pattern and that serves as the input of upper layer. In so doing, MNFL not only penalizes erroneous input but also mitigates the cascading effect of wrong information to other nodes in the upper layer. In our experiments, MNFL shows better accuracy even when all relevant information is given (Noise0 set), but its real strength is the robustness against erroneous input patterns. The accuracy of MNFL is maintained at the highest level among the three competitors and maintains 72% of the accuracy when two out of five input patterns are less relevant. Since it is very difficult for casual owners to observe the abnormality of their dogs correctly, it is expected that the input patterns include irrelevant information.

Since our goal in developing this pre-diagnosis system is not to compete with a veterinarian’s diagnosis but to alert casual users to monitor the abnormal behavior of their dogs, coping with such abnormalities as early as possible, this MNFL with sufficiently high robustness and good accuracy showcased in this experiment encourages us to make our system more applicable in real-world application.

## 5. Conclusions

As the pet dog healthcare market is expanding rapidly in Korea, casual pet owners want to have more reliable information about strategies to detect their pet’s abnormal behavior and appropriate coping methods. In this paper, we propose a neuro-fuzzy learning-based dog disease pre-diagnosis system. Such a system should be robust against noise input, since casual pet owners frequently give incorrect/irrelevant information to the system.

First, we set up the symptom–disease database based on several textbooks with veterinarians’ guidance. Then, we tested three fuzzy inference algorithms designed for our application. PFCM-R is good when almost all input symptoms are relevant, but its performance drops fast when outlier/noise information increases. Hybrid association (FHAL) is weakly robust, but its inference accuracy is limited. We verified that the multi-layered neuro-fuzzy learner (MNFL), designed for this paper, successfully filters input relevance with a fuzzy clustering strategy and carefully designed feed-forward network structure, which associates symptoms and target diseases and works well against noisy input patterns.

The limitation of this paper is that the result of this experiment is not comparable to a result with the accuracy of a diagnosis based on clinical data. The experiment only verifies the behavioral pattern of three fuzzy algorithms against noisy input and the power of robustness in inference.

In the future, the system should extend the database, covering more diseases and symptoms that are not covered by introductory textbooks. To do this, we require deeper collaboration with veterinarians, and the database and inference engines should be tested under real-world clinical data, considering factors such as the effects of breed, aging, etc.

## Figures and Tables

**Table 1:** Example of disease–symptom association.

SymptomT Table			DiseaseT Table			
**SymptomID**	**Description**	**ObservedPart**	**DiseaseID**	**Description**		**AppearedPart**
1	shedding tears	3	1	glaucoma		3
2	redness of eggwhite	3	2	dermatomycosis		1
3	itch	1	3	pulmonitis		10
…	…	…	…	…		…
217	cough	14	150	hepatic encephalopathy		12
**ObservedPart Table**		**AppearedPart Table**		**Association Table**		
**Part#**	**Description**	**Part#**	**Description**	**ID**	**DiseaseID**	**SymptomID**
1	full body	1	wound/skin	1	1	1
2	head	2	oral	2	1	2
3	eye	3	eye	3	2	1
4	skin	4	tail	4	2	3
…	…	…	…	…	…	…
16	faces	12	circulatory system	467	150	34

**Table 2:** Inference accuracy over noise level.

Accuracy	MNFL	FHAL	PFCM-R
Noise0	94%	82%	90%
Noise1	84%	78%	80%
Noise2	72%	74%	54%
Noise3	54%	46%	22%

**Table 3:** Inference within Top3 over noise level.

Top3	MNFL	FHAL	PFCM-R
Noise 0	98%	90%	100%
Noise 1	92%	82%	96%
Noise 2	80%	80%	80%
Noise 3	66%	52%	52%

## Data Availability

The data presented in this study are available on request from the corresponding author. The data are not publicly available due to Institutional regulations.
